# Role of Simulations in the Treatment Planning of Radiofrequency Hyperthermia Therapy in Clinics

**DOI:** 10.1155/2019/9685476

**Published:** 2019-08-29

**Authors:** Bibin Prasad, Jung Kyung Kim, Suzy Kim

**Affiliations:** ^1^Department of Radiation Oncology, SMG-Seoul National University Boramae Medical Center, Seoul 07061, Republic of Korea; ^2^Department of Radiology, UT Southwestern Medical Center, Dallas, TX 75390-8542, USA; ^3^School of Mechanical Engineering and Department of Integrative Biomedical Science and Engineering, Graduate School, Kookmin University, Seoul 02707, Republic of Korea

## Abstract

Hyperthermia therapy is a treatment modality in which tumor temperatures are elevated to higher temperatures to cause damage to cancerous tissues. Numerical simulations are integral in the development of hyperthermia treatment systems and in clinical treatment planning. In this study, simulations in radiofrequency hyperthermia therapy are reviewed in terms of their technical development and clinical aspects for effective clinical use. This review offers an overview of mathematical models and the importance of tissue properties; locoregional mild hyperthermia therapy, including phantom and realistic human anatomy models; phase array systems; tissue damage; thermal dose analysis; and thermoradiotherapy planning. This review details the improvements in numerical approaches in treatment planning and their application for effective clinical use. Furthermore, the modeling of thermoradiotherapy planning, which can be integrated with radiotherapy to provide combined hyperthermia and radiotherapy treatment planning strategies, are also discussed. This review may contribute to the effective development of thermoradiotherapy planning in clinics.

## 1. Introduction

Hyperthermia therapy is used as an adjunctive cancer treatment modality in which the temperature of cancerous tissues is increased to enhance the clinical efficacy of chemotherapy and radiotherapy [1–4]. Radiofrequency (RF) generator with electrode/antenna is a technique through which hyperthermia therapy can be achieved. RF (100 kHz–3 GHz) is a form of electromagnetic (EM) energy that comprises an alternating electric current, and this EM energy is absorbed when passing through the human body to target tumor tissues as tumors have different dielectric properties compared with those of surrounding normal tissues [5]. During hyperthermia treatment, the tumor temperature is increased to 40°C–45°C for 30–60 min for effective radio and chemosensitization and the blood perfusion is increased [6, 7].

Hyperthermia treatment planning (HTP) includes the selection of an optimal treatment technique that is in current use and for which several tools and techniques have been developed. Previous review articles have shown that HTP is an essential approach and has a pivotal role in the control, improvement, and assessment of hyperthermia treatment quality [6–8]. HTP, which involves obtaining patient data and calculating power deposition and temperature distribution in tissues, enables improvements in treatment quality [8]. To provide a complete 3D distribution of temperature in the human body, treatment planning must consider complex heating systems, human anatomy, dielectric tissue properties, thermophysical changes in tissues in response to heat and physiology, especially blood perfusion [9]. For clinical applications, high-resolution patient imaging, specific absorption rate (SAR) and thermal models, evaluation software, and data acquisition systems are available these days [7].

To improve treatment quality, simulation techniques have been extensively used in clinics, and HTP techniques have been developed for clinical use [10–13]. An HTP system called HyperPlan was assessed for its clinical practicability in predicting the toxicity and efficacy of hyperthermia treatment [14]. VEDO (visualization tool for electromagnetic dosimetry and optimization) is an HTP software tool developed for quantitative optimization of SAR in clinical head and neck cancer treatment [11]. Clinical trials have been conducted to verify HTP calculations by measuring the SAR in 11 patients with uterine cervical cancer using the 70-MHz Academic Medical Center (AMC)-4 waveguide system [15]. Another clinical trial assessed the feasibility of HGS (online HTP-guided steering) in improving the treatment quality for locally advanced cervical cancer [10].

**Figure 1:**
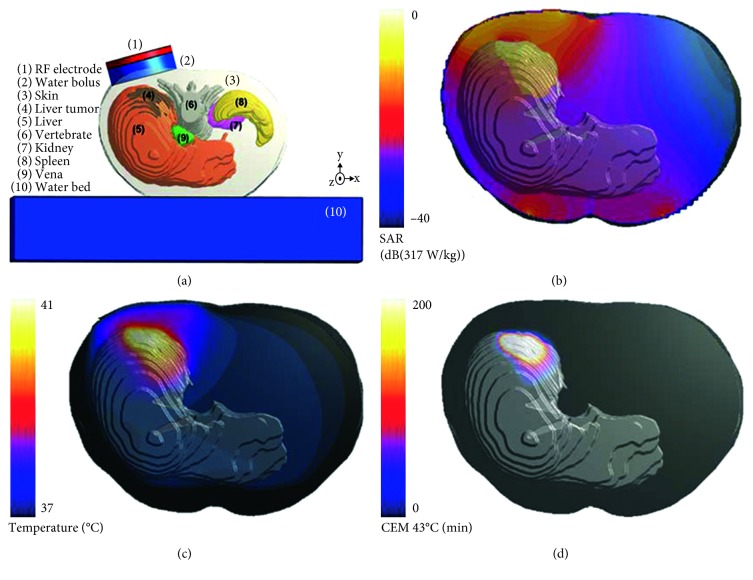
Computational modeling and analysis using a patient-specific liver tumor model: (a) 3D human anatomy with an RF system; (b) SAR distribution; (c) temperature distribution; (d) thermal dose.

Commercial software packages are available for HTP. The most widely used simulation software for locoregional hyperthermia treatment is Sigma HyperPlan (Dr. Sennewald Medizintechnik GmbH, Munchen, Germany), which was developed by the Konrad Zuse Institute; however, this software is limited to modeling locoregional hyperthermia systems. More flexible treatment planning systems have been developed, including the AMC DIVA HTP system (AMC, Amsterdam, Netherlands), SEMCAD X (SPAEG, Zurich, Switzerland), and Sim4Life (ZMT, Zurich, Switzerland), which include additional and advanced treatment planning tools such as animal and human phantom models, tissue models, magnetic resonance imaging (MRI) modules, and dispersive fitting modules. Besides these specialized HTP tools, general commercial software, including COMSOL Multiphysics (http://www.comsol.com), CST Studio Suite (http://www.cst.com), and Ansys (http://www.ansys.com), can also be used for calculating EM and temperature distributions.  Figure 1 presents the 3D modeling, SAR distribution, temperature distribution, and thermal dose calculations in a patient-specific human model simulated with Sim4Life software [5].

This review summarizes the various technical aspects in radiofrequency hyperthermia, including mathematical models, tissue properties, phantom studies, realistic human anatomy, tissue damage and thermal dose, and thermoradiotherapy planning.

## 2. Numerical Modeling

A summary of the major mathematical models used for HTP is detailed below. The EM field distribution during RF heating can be calculated using Maxwell's equation. The finite-difference time-domain (FDTD) method can be used for a wide range of frequencies (250 Hz–3 GHz) [9, 16–25]:

(1) $$\begin{array}{c} \varepsilon \frac{\delta E}{\delta t}+J=\nabla H-\frac{\delta B}{\delta t}=\nabla E, \end{array}$$

(2) $$\begin{array}{c} \frac{\partial E_{z}}{\partial t}=\frac{1}{\varepsilon }\left(\frac{\partial H_{y}}{\partial x}-\frac{\partial H_{x}}{\partial y}-\sigma E_{z}\right), \end{array}$$

where *ε* is the relative permittivity, *E* is the electric field strength (V·m^−1^), *J* is the current density (A·m^−2^), *H* is the magnetic field strength (A·m^−1^), *B* is the magnetic flux density (T), and *σ* is the electrical conductivity (S·m^−1^).

The vector equations in equation (1) are written as six separate partial differential equations, one of which is shown as equation (2) [25].

For capacitive heating, quasistatic approximation is used to obtain the potential distribution [26–29]:

(3) $$\begin{array}{c} \nabla \left(-\varepsilon \nabla \phi \right)=0,\\ E=-\nabla \phi , \end{array}$$

where *ϕ* is the electric potential (V).

The E-field calculation from the FDTD and quasistatic approaches enables the calculation of the SAR in tissues [29]:

(4) $$\begin{array}{c} \text{SAR}=\frac{\sigma }{2\rho }{\left|E\right|}^{2},\\ Q_{\text{r}}=\rho \text{SAR}=\frac{\sigma }{2}{\left|E\right|}^{2}, \end{array}$$

where *ρ* is the mass density (kg·m^−3^), SAR is the specific absorption rate (W·kg^−1^), and *Q*_r_ is the EM power absorbed (W·m^−3^).

The RF energy obtained from the EM simulation is given as a heat source (*Q*_r_) to the Pennes bioheat model to calculate the temperature distribution [30–32]:

(5) $$\begin{array}{c} \rho c\frac{\partial T}{\partial t}=\nabla \cdot \left(k\nabla T\right)-\omega_{\text{b}}c_{\text{b}}\left(T-T_{\text{b}}\right)+Q_{\text{r}}+Q_{\text{m}}, \end{array}$$

where *c* is the specific heat (J·kg^−1^·K^−1^); *T* is the temperature (K); *t* is the time (s); *k* is the thermal conductivity (W·m^−1^·K^−1^); *Q*_m_ is the metabolic heat generation rate (W·m^3^); *ω*_b_ is the blood perfusion rate (kg·m^−3^·s^−1^); and *c*_b_ and *T*_b_ correspond to the specific heat and temperature of blood, respectively.

Besides the temperature distribution, thermal dose can be obtained using the cumulative equivalent minutes (CEM at 43°C) [33]:

(6) $$\begin{array}{c} \text{CEM}43=\int_{t_{0}}^{t_{\text{final}}}R^{43-T\left(t\right)}dt, \end{array}$$

where *R* is the temperature dependence of the rate of cell death (*R* = 0.5 for *T* > 43°C, *R* = 0.25 for 39°C ≥ *T* ≥ 43°C, and *R* = 0 for *T* < 39°C), *dt* is the time interval with which temperature measurements are made (min), and *t*_o_ (min) and *t*_final_ (min) are the initial and final heating periods, respectively.

The combined therapeutic outcomes of radiotherapy and hyperthermia can be calculated in terms of the equivalent radiation dose as follows [34]:

(7) $$\begin{array}{c} {\text{EQD}}_{RT}=\frac{\alpha \left(T\right)D+G\beta D^{2}}{\alpha +\beta d}, \end{array}$$

where *α* (Gy^−1^) and *β* (Gy^−2^) are the radiosensitivity parameters at 37°C, *α*(*T*) is the temperature-dependency of *α*, *d* is the fractionated dose (Gy), *D* is the total radiation dose (Gy), and *G* is the Lea–Catcheside protraction factor.

## 3. Tissue Properties

Tissue properties are important parameters in simulations in determining the temperature distribution of the target and its surrounding tissues. Electrical conductivity, relative permittivity, density, thermal conductivity, specific heat, metabolic heat generation rate, and blood perfusion rate are the major tissue properties associated with the electrothermal simulations of mild and ablative hyperthermia [35]. Several studies have been conducted for the measurement of normal tissue properties, and a wide database is available [36–39]. The most complete database for tissue properties is available at IT'IS Foundation (itis.swiss/database) [40]. In RF thermal therapy, the dielectric properties (electrical conductivity and relative permittivity) are the most important properties in calculating energy deposition in tissues. Previous studies have reported that the dielectric properties of tissues vary as the frequency increases [36–38]. Tumors present a very wide distribution of electrical properties [41]. Generally, tumor tissues exhibit a high dielectric property compared with that exhibited by normal tissues; however, certain tumors exhibit low dielectric properties compared with that exhibited by normal tissues [39, 42–44]. Studies have also reported that there are variations in tumor properties in different patients and different cancer stages and that these effects required further evaluations [45, 46]. A simulation study showed that calculations with different dielectric properties lead to different SAR, temperatures, and thermal dose calculations [29]. Tables 1 and 2 summarize the various tumor phantom/tissue properties considered in simulation studies at different frequencies.

Some of the properties displayed in Tables 1 and 2 may be based on certain assumptions such as high/low electrical conductivity, relative permittivity, and blood perfusion. However, certain tumor property measurements have been reported based on human tumor samples, [39, 45, 46, 59, 60], which will be more correct to use in simulations, thereby providing more accurate SAR/temperature distributions. The temperature-dependency of electrical and thermal properties must be considered important for hyperthermia and ablative temperature treatment planning to enhance the accuracy of treatments [35, 61, 62]. In the case of thermal conductivity, the change correlates directly with temperature, whereas for blood perfusion, a strong irreversible component is observed with the interaction with time and temperature. There will be an increase in blood perfusion at mild hyperthermia temperatures and collapse in blood flow at ablative temperatures. For dielectric properties at RF frequencies, the tissue state, such as in vivo, in situ, and ex vivo, with time after tissue extraction has a significant impact on dielectric property measurement, which needs to be considered [35].

## 4. Locoregional Hyperthermia Therapy

Locoregional therapy is usually applied at hyperthermic temperature of 40°C–45°C. Studies with tissue-mimicking phantom models and 3D human anatomy models have been conducted to demonstrate its efficacy in the treatment of various types of cancer as summarized below.

### 4.1. Tissue-Mimicking Phantom Models

Tissue-mimicking phantoms, the physical properties of which are close to human tissues, are important in the development of experimental and numerical techniques. Many studies have been performed to develop phantoms with dielectric and thermal properties similar to those of human tissues [63–74]. An agar phantom that is suitable for use in simulations was developed in the 5–40 MHz range. This type of phantom has distinct advantages, including the ability to vary its dielectric properties, retain its original shape, and avoid deorientation by molding for one year [63]. Cylindrical phantoms are commonly used to investigate various RF hyperthermia mechanisms, and selective and deep heating characteristics have been evaluated with cylindrical phantoms experimentally and numerically for 13.56 MHz RF hyperthermia systems [47]. Simulations and experiments have been performed using a cylindrical phantom with fat and muscle layers to determine the effect of large vessel counterflow on temperature distribution by hyperthermia [75]. A computational phantom comprising fat, muscle, and tumor tissues has been used for temperature prediction validation in the development of a temperature-based feedback control system for an EM-phased array hyperthermia system [51]. Simulations on a cylindrical split phantom have also comprised vegetable oil and saline with dielectric properties similar to those of human tissues for B_1+_ imaging to validate SAR models for RF hyperthermia [76]. SAR distribution for centrally located targets in tissue-equivalent phantoms has been simulated, with optimal distributions established at 140 MHz [13]. The size of SAR hot spots was reduced at higher frequencies when simulations were performed in the range of 64–600 MHz [77]. Temperature increase due to SAR from RF fields has been numerically and experimentally validated with phantom models that provide good agreement and demonstrate the possibility of using simulations to ensure safety during MRI [52]. The feasibility of using nanoparticles with RF systems has been reported with phantom models to increase tissue temperature in localized and deep-seated tumor regions [49, 50, 77]. The addition of magnetic nanoparticles to regions drew more heat than that by surrounding regions in both simulations and MR thermometry [50], and a two-channel RF system exhibited superior capability in localizing heat to the nanoparticle-mediated tumor region compared with that in the normal region [77].

### 4.2. Realistic Human Anatomy Models

The accurate prediction of SAR and temperature distribution in human tissues is necessary for the effective use of RF-induced hyperthermia in clinics. By using patient-specific computational models, HTP quality can be improved. Numerical simulations with phantom and human anatomy models can evaluate the efficacy of RF techniques developed for clinical use. A 3D model derived from human anatomical data showed that simulations can assist in the clinical use of RF applicators to heat the region of the thorax [78]. In patients with malignant brain tumors, computer simulations showed good agreement with measurements for interstitial hyperthermia [79, 80]. Temperature measurement and simulations in 11 human subjects with intracranial tumors showed a difference of approximately ±0.75°C [79], and a difference of only 0.4°C was obtained in 4 patients with malignant glioma [80]. The RF EM field patterns obtained from simulations in human anatomies was verified using the MR B_1+_ imaging technique with an accuracy of 3.5%, suggesting that the MR B_1+_ imaging technique is a valuable technique for obtaining the dielectric interaction between human anatomy and the RF field [81]. MR B_1+_ is an experimentally validated technique for hyperthermia SAR treatment planning [76]. Given that SAR is a major parameter in MRI safety regulation [82], patient-specific local SAR determination with MRI measurements and simulation yields accurate estimation of SAR for the brain [82]. The efficacy of RF hyperthermia therapy can be further improved by incorporating MR B_1+_ imaging and SAR determination technique [81, 82] with necessary modifications in HTP. Minimally invasive 3D dosimetry based on patient-specific simulations and sensory feedback was found to be feasible for use in hyperthermia therapy. The prediction of the T50 treatment parameter had a median accuracy of 0.4°C with patient-specific properties [56]. Patient-specific simulations in the treatment of liver cancer showed its capability to heat tumors with 13.56 MHz RF hyperthermia selectively and demonstrated the effect of electrode size and power modulation in predicting temperature distribution and thermal dose for effective clinical treatment planning [5].

Among RF hyperthermia systems, RF phased array applicators are effectively used in clinics for the selective heating of tumor tissues while sparing normal healthy tissues [83]. There have been several developments in system design to improve its efficacy in clinical use. A considerable improvement in the focusing of SAR distribution in tumor regions was observed when parameters, such as frequency (100–120 MHz), bolus chamber (tapered), dipole length (17–30 cm), and phase and amplitude of power to various dipoles, were adjusted in numerical calculation using an annular phase array of eight dipole antennas coupled with water bolus on human anatomy [84]. Furthermore, when the previous validation of RF hyperthermia array modeling with EM field distribution measurement considered variations in bolus size and waveguide quantity, the accuracy was found to be inadequate for quantitative SAR dosimetry for individual patients [85]. The technological development of RF phased array hyperthermia has been achieved by developing a treatment planning program. Improvements in the index temperature of 1°C–2°C were achieved when the single ring was upgraded to a triple ring with free-phase selection for individual antenna or as pairs [86]. Temperature-based optimization for HTP with high resolution was implemented to maximize tumor temperature using E-field distributions as a primary input. The method was found to work well in clinical situations [53]. A prospective HTP together with high-resolution temperature optimization improved the treatment in 16 patients with esophageal cancer [87]. A useful tool for optimizing clinical treatments was provided by advancing the simulation accuracy with temperature-dependent blood perfusion and noninvasive thermometry with thermal imaging. The quality of hyperthermia treatment can be improved by performing simulations routinely as part of pretreatment planning in clinics [88]. For the planning, evaluation, and optimization of hyperthermia treatments, the flexible software package Plan2Heat was developed. Plan2Heat performs EM and temperature simulations for RF and other treatment techniques. The package also enables the sophisticated thermal modeling of 3D vasculature using a vessel generation algorithm. The source code of the software allows the easy extension of the software package for future applications [26].

A potential hyperthermia-phased array treatment system for head and neck cancer has been investigated and developed successfully with the assistance of numerical simulations that consider various parameters [89], and an efficient treatment system HYPERcollar was developed with an antenna ring, water bolus, and a positioning system [57]. Numerical simulations enhanced the stability, water bolus shape, skin contact, and positioning of patients (±5 mm), improving patient treatment quality, reproducibility, operator handling, and patient safety [90]. In a study of 27 patients, pretreatment planning involved the use of 3D human models to determine the SAR target level of 25% and to decide whether hyperthermia treatment was possible in patients. The study confirmed the feasibility and safety of hyperthermia therapy with promising outcomes and good compliance [91]. To further investigate the treatment quality of HYPERcollar, comparisons were made with planar applicators, including the lucite cone applicator (LCA) and current sheet applicator (CSA), in 24 patients with a clinical target volume of up to 6 cm from the surface. Simulations revealed that HYPERcollar was the preferred system over the LCA and CSA [58].

Water bolus temperature influences the prediction of tissue temperature in hyperthermia therapy. 3D EM and thermal simulations were performed to develop a guideline for water bolus temperature for the LCA [92]. A convective coefficient for the skin and water bolus surface was used, and convective coefficients of different water boluses were measured (75–125 W·m^−2^·K^−1^). The 3D model consisting LCA, water bolus, and a block of tissue was evaluated by comparing simulations with clinical measurements, and the temperature distribution of tissues was predicted well on a global view, but some temperature probes showed a deviation of 1.5°C–2.0°C [92]. Water bolus temperature was also assumed to be constant in hyperthermia simulations. In a patient-specific simulation of liver cancer treatment with RF hyperthermia, the water bolus temperature was kept constant at 25°C to keep the surface cool to prevent skin burning [5]. Moreover, in capacitive hyperthermia treatment for lung cancer using a human thorax model, the water bolus surrounding the thorax region is maintained at a constant temperature [93]. In HYPERcollar systems for head and neck hyperthermia, the water bolus shape has a significant impact. With high reproducibility, water bolus can reduce the risk of underexposure and nearly the whole head and neck of the patient can be enclosed with surrounding water bolus, improving the treatment quality [58, 90, 94].

## 5. Estimation of Thermal Tissue Damage

The thermal dose (cumulative equivalent minutes at 43°C, CEM43) model is mainly used to calculate tissue damage during hyperthermia therapy [33]. Thermal thresholds for tissue damage have been investigated, and preclinical and clinical examinations have been conducted to assess the tissue damage in various types of tissues [95–103]. By using optimized time-dependent temperature distribution in tumor tissues, tissue damage can be preplanned to predict clinical outcomes [29]. The effect of thermal wave characteristics on thermal dose distribution was numerically evaluated during thermal therapy. For total deposited energy, the time lag of the peak temperature became pronounced, and the peak level was decreased with increasing relaxation time. No significant difference was observed in thermal dose distribution with or without the effect of thermal relaxation time [104]. To determine the safety of RF-induced local thermal hot spots using a 1.5-T body coil, simulations were performed on four anatomical models of varying sizes and shapes, and the results suggested that a thermal dose is recommended rather than SAR or temperatures to ensure safety [105]. SAR thresholds were quantified for electromagnetic exposure using functional thermal dose limits. From the literature, the lowest thermal dose required to induce acute local tissue damage was used to calculate the corresponding thermal dose functional SAR limits. The results were evaluated by comparing the calculations of a real human anatomy simulation for head and neck hyperthermia treatment. It was found that for muscle tissues, the current restriction on 10 g peak spatial average SAR can reach up to 31.2 W/kg. Therefore, they suggested exposure-specific guidelines (i.e., exposure-specific SAR limits) rather than the current generic guidelines for safety [106]. Thermal dose calculations in a patient-specific simulation study for liver cancer treatment also predicted that no damage occurs at normal tissues while selectively increasing the tumor temperature, indicating that it is a promising technique for HTP [5]. In HTP, tumor property variations may also require consideration for precise tissue damage calculations. When thermal dose simulations were compared with dielectric properties measured versus dielectric properties obtained from a previous study using a mouse tumor model, accurate thermal dose calculations were obtained when the measured dielectric properties of the tumor were used in simulations [29].

## 6. Thermoradiotherapy Planning

Substantial prospective and randomized clinical data show the clear benefits of combined hyperthermia and radiotherapy for various types of cancer [107]. A method for estimating the therapeutic effect of radiation and hyperthermia can be numerically quantified in terms of equivalent radiation dose. The temperature-dependent parameters *α* and *β* of the linear-quadratic (LQ) model can effectively express radiosensitization by hyperthermia [54]. The LQ parameters of human tumors can be useful for the prediction of radiobiological response in clinical radiotherapy [108]; however, few studies have reported the LQ parameters at hyperthermic temperatures [109, 110]. Numerical models were developed to calculate equivalent radiation dose and analysis for patients with prostate and cervical cancers, and these models revealed an escalation in equivalent radiation dose [54, 55]. For the planned average minimum, mean, and maximum tumor temperatures (40.5°C, 41.6°C, and 42.4°C, respectively) and the radiation therapy doses of 62.9, 76, and 81 Gy, respectively, the equivalent radiation doses increased to 70.3, 86.3, and 93.6 Gy, respectively, with an extended isodose level of 95% [54] in patients with prostate cancer. Biological modeling to quantify radiosensitization in patients with cervical cancer in terms of equivalent dose for combined radiotherapy and hyperthermia resulted in an increase in the radiation dose from 7.3 to 11.9 Gy compared with that for radiotherapy alone [55]. Equivalent radiation dose calculation with RF hyperthermia for human lung cancer xenografts using A549 and NCI-H1299 cell lines showed that a higher radiation dose escalation was observed for NCI-H1299 compared with that observed for A549 (36.07 Gy for A549 cells and 39.66 Gy for NCI-H1299 cells from 20 Gy) [111].

The X-Term software package was developed to calculate the equivalent radiation dose using an extended version of the LQ model. The software can support decision-making for optimal clinical treatment, enabling the biological evaluation of thermoradiotherapy plans via equivalent 3D radiation dose distributions [34]. It is important that planning combines both radiation and hyperthermia therapy by considering the synergistic actions of the two modalities. The first planning tool to consider the inhibition of DNA damage repair was developed to model the combined effect of radiation and hyperthermia therapy. The outcomes suggested the further realization of radiobiological data for different types of tumors, temperatures, sequences, and oxygenation conditions, in addition to direct cell death and reoxygenation [112]. To improve patient selection and treatment plan optimization in thermoradiotherapy, a biological model as a function of variables was developed to predict treatment outcomes. The model describes cell survival dependency on dose, temperature, and time interval [113]. The effect of the time interval between radiotherapy and hyperthermia was evaluated to determine the therapeutic advantage of using biological modeling. The study demonstrated that the highest therapeutic gain for patients with cervical cancer was obtained in thermoradiotherapy when hyperthermia was immediately applied prior to or following radiation treatment [114].

## 7. Future Perspectives

**Figure 2:**
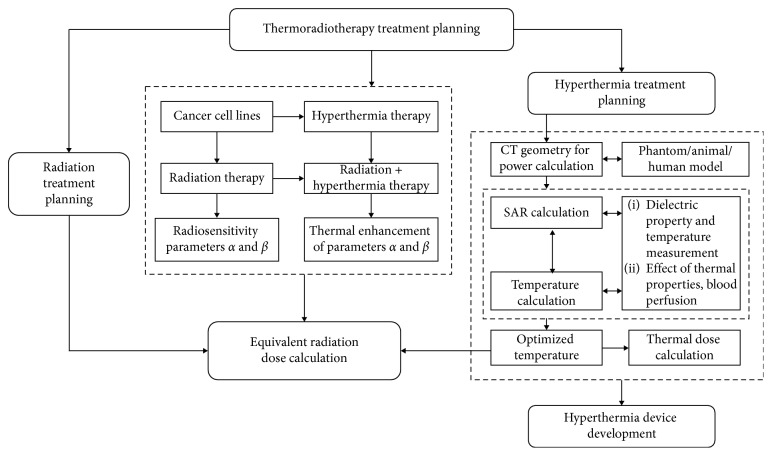
Thermoradiotherapy treatment planning for effective radiation treatment planning, HTP, equivalent radiation dose implementation, and hyperthermia treatment device development.

For the effective clinical application of HTP, temperature-dependent blood perfusion and the dielectric properties of tumor tissues that consider variations according to patients and tumor stages require further investigations for various types of cancer. This may be investigated with the development of techniques, such as magnetic resonance electrical properties tomography (MREPT) and dictionary-based electric properties tomography (dbEPT) for measuring dielectric properties [115, 116] and high-frequency Doppler ultrasound and indocyanine green imaging for measuring blood perfusion [117, 118]. Although discrete vasculature (DIVA) models can provide sophisticated 3D vessel networks [119, 120] and software to model incomplete discrete vessel for the DIVA model from CT or MR angiography is available [121, 122], further developments and validations may need to obtain precise patient-specific temperature distribution in the vessel structures. For effective thermoradiotherapy planning based on equivalent radiation dose escalation, the determination of radiosensatization by hyperthermia on different cancer cell lines is required to apply this approach to various types of cancer. It may result in a treatment planning platform for combined hyperthermia and radiotherapy with high tumor control probability. To achieve this, improvements and updates in the theoretical framework and software tools require further development and validation. The development of a thermoradiotherapy planning strategy by integrating multiple treatment modalities may be an efficient way to enhance radiation treatment planning, HTP, equivalent radiation dose, and effective device development for combined radiation and hyperthermia therapy (Figure 2).

## 8. Conclusions

Studies have demonstrated the importance of simulations in the development of effective HTP techniques for clinical use. Dielectric properties and variations in blood perfusion are important in calculating energy deposition and temperature distribution in target tissues. Based on the accurate numerical modeling for hyperthermia treatment, efficient hyperthermia devices can be developed and the limitations of existing systems can be improved. Tissue damage and thermal dose calculations can predict the hot spots induced in applying RF energy, ensuring the safety and visualization of the lesion volume prior to treatment. Biological modeling for the calculation of an equivalent radiation dose may be effective for the quantification of thermoradiotherapy planning for combined hyperthermia and radiotherapy in clinics. The appropriate modeling and simulation of thermoradiotherapy is likely to contribute to the prediction of the efficacy and toxicity of cancer thermal therapy.

## Figures and Tables

**Table 1:** Dielectric and thermal properties of tumor phantom used in simulations at different frequencies from the literature.

Frequency (MHz)	Tumor phantom type	Density (kg·m^−3^)	Relative permittivity	Electrical conductivity (S·m^−1^)	Specific heat (J·kg^−1^·K^−1^)	Thermal conductivity (W·m^−1^·K^−1^)	Reference
13.56	Egg white	1131	66	0.705	2679	0.393	Hossain et al. [47]
26	Agarose	1020	79.86	0.6	4267	0.555	Kim et al. [49]
36	Agarose	1020	80	1.38	4267	0.555	Kim et al. [50]
110	Agar	900	80	0.70	3500	0.210	Kowalski and Jin [51]
165	Agar	1021	74.15	2.97	4200	0.498	Oh et al. [52]

**Table 2:** Dielectric and thermal properties of different cancer used in simulations at different frequencies from the literature.

Frequency (MHz)	Cancer type	Density (kg·m^−3^)	Relative permittivity	Electrical conductivity (S·m^−1^)	Specific heat (J·kg^−1^·K^−1^)	Thermal conductivity (W·m^−1^·K^−1^)	Blood perfusion (kg·m^−3^·s^−1^)	Reference
13.56	Liver	1078.75	70	0.5	3763	0.5191	2.15	Prasad et al. [5]
13.56	Muscle	1070	278.85	0.7847	3421.2	0.4949	0.35	Prasad et al. [29]
70	Cervical, pancreas, Esophagus, Prostate	1050	65	0.74	3639	0.56	1.8	Kok et al. [13, 26, 53, 54], Crezee et al. [55]
100	Cervical	1050	58	0.78	3639	0.56	1.8	Kok et al. [13]
120	Cervical	1050	55	0.80	3639	0.56	1.8	Kok et al. [13]
130	Cervical	1050	54	0.81	3639	0.56	1.8	Kok et al. [13]
140	Cervical	1050	53	0.82	3639	0.56	1.8	Kok et al. [13]
150	Cervical	1050	52	0.83	3639	0.56	1.8	Kok et al. [13]
434	Head and neck	1050	59.0	0.89	3950	0.51	7.35, 1.32^*∗*^/15.58^*∗*^	Verhaart et al. [56], Paulides et al. [57], Drizdal et al. [58]^*∗*^
